# Design and Implementation of a Smart Traffic Signal Control System for Smart City Applications

**DOI:** 10.3390/s20020508

**Published:** 2020-01-16

**Authors:** Wei-Hsun Lee, Chi-Yi Chiu

**Affiliations:** 1 Department of Transportation and Communication Management Science, National Cheng Kung University, Tainan 701, Taiwan; 2 Institute of Telecommunication Management, National Cheng Kung University, Tainan 701, Taiwan; r96064013@mail.ncku.edu.tw

**Keywords:** emergency vehicle signal preemption, vehicular network, adaptive traffic signal control, transport signal priority, smart city infrastructure

## Abstract

Infrastructure supporting vehicular network (V2X) capability is the key factor to the success of smart city because it enables many smart transportation services. In order to reduce the traffic congestion and improve the public transport efficiency, many intelligent transportation systems (ITS) need to be developed. In this paper, a smart traffic signal control (STSC) system is designed and implemented, it supports several smart city transportation applications including emergency vehicle signal preemption (EVSP), public transport signal priority (TSP), adaptive traffic signal control (ATSC), eco-driving supporting, and message broadcasting. The roadside unit (RSU) controller is the core of the proposed STSC system, where the system architecture, middleware, control algorithms, and peripheral modules are detailed discussed in this paper. It is compatible with existed traffic signal controller so that it can be fast and cost−effectively deployed. A new traffic signal scheme is specially designed for the EVSP scenario, it can inform all the drivers near the intersection regarding which direction the emergency vehicle (EV) is approaching, smoothing the traffic flow, and enhancing the safety. EVSP scenario and the related control algorithms are implemented in this work; integration test and field test are performed to demonstrate the STSC system.

## 1. Introduction

The population in the world has reached 7.7 billion in 2019, an increase of 1 billion since 2007, and the size of the global population would stand between 8.5 and 8.6 billions in 2030 [1]. Population growth causes an increase in the number of vehicles, and a lack of effective traffic management would lead to huge economic lost such as energy consumption, greenhouse gas emissions, and time lost. For example, the United States lost 5.5 billion hours of time due to traffic congestion from 2000 to 2010 [2]. With the continuous population growth, traffic congestion has become one of the biggest obstacles restricting the city economic development, it results in high consumptions of fuel, increases the cost of commutes, and also pollutes the environment.

In order to reduce the traffic congestion and improve the public transport efficiency, infrastructures which enables smart transport services would be the key factor for the success of intelligent transportation systems (ITS). For example, day-1 and day-1.5 services proposed in the cooperative ITS (C-ITS) in H-2020 [3] demonstrate a trend in the development of ITS. The development of cooperative intelligent transportation systems, such as transit signal priority (TSP) for public transport, emergency vehicle signal preemption (EVSP), and adaptive traffic signal control (ATSC), is considered as the one of the important indexes in smart and sustainable cities. Among these services, the key technology is vehicular network, which enables vehicles equipped with an on-board unit (OBU) exchange real-time information with roadside unit (RSU) and other OBUs, achieving the communication scheme of vehicle-to-everything (V2X). Two vehicular network communication protocols are available including 802.11p [4] and LTE-V2X [5], which are defined by IEEE and 3GPP associations, respectively.

A traffic signal controller, as the most important part of infrastructure in smart city transportation, is the main coordinator for the urban traffic flows. Traditionally, three types of traffic control strategies are adopted including pre-time (predefined signal plan), actuated (triggered signal control), or ATSC (adaptive traffic signal control) depending on the traffic assignment strategy decided by traffic management authority. However, these approaches do not fulfil the intelligent transportation systems requirements in the smart city, such as V2X-based applications proposed in a C-ITS project.

In this paper, an integrated RSU for smart traffic control system (STSC) is designed and implemented to support multi-modal V2X-based applications including emergency vehicle signal preemption, transit signal priority system, adaptive traffic signal control, and pre-time signal control, eco-driving supporting, road work, and incident alarm message broadcasting. The features of the STSC system are the integration of multi-modal applications, modular design, real-time traffic control algorithms, and traffic data collection. The proposed STSC system is designed by a unified system architecture with an RSU middleware which organized by modular functional design. Moreover, it is backward compatible with traditional traffic signal controller so that these applications can be fast and cost efficiently deployed without replacing legacy infrastructure.

The remainder of this paper is organized as follows. Background knowledge of ITS applications and related works discussion are illustrated in Section 2. The details of the proposed STSC system and algorithms are presented in Section 3. System implementation and experiment results are illustrated in Section 4. Finally, the concluding remarks and some suggestions for future works are presented in Section 5.

## 2. Related Works on ITS Applications

### 2.1. Emergency Vehicle Signal Preemption (EVSP)

More than 98% of respondents have encountered ambulances on public roads in Dearborn Heights, but 82.9% of the respondents had at least one experience of failing to respond appropriately when emergency vehicles (EV) approached [6]. The paper pointed out that, in the United States between 2004 and 2008, there were 3708 accidents involving emergency vehicles. After the in-vehicle information system (such as on-board unit, smart phone, radio), the interference to the general driving is more serious, which will cause the driver to have a slower response to the emergency vehicle. Emergency vehicles need to respond quickly to save the lives and property. In some emergency situations, early response is very important to affect life and death in a few seconds. Every one minute of delay in the patient′s first aid, the chance of survival would be reduced by 7–10%. For a fire accident, half a minute for each delay will double the fire. For traffic accidents, one third of the deaths associated with vehicle accidents can be prevented by ensuring faster rescue of emergency vehicles, so rapid rescue is already a very urgent and important matter [7].

In order to reduce EV rescue delay, Savolainen et al. use LED equipment to display the signs of emergency vehicles [6]. When an EV approaches the intersection, the OBU in EV will communicate with the signal controller. The traffic signal controller then turns on the emergency vehicle LED flashing, the passersby can react early to the emergency vehicle approach and then prepare for the early arrival. However, EV drivers still have to slow down when driving through the intersection due to the signal controller not supporting emergency vehicle signal preemption.

In [7], an EVSP system was proposed to solve this problem by extending the green period or cutting off the red period to facilitate the EV passing through the intersection. It assigns different weights to different types of EV, such as the ambulance, fire engine, and police vehicles, so that the signal controller can serve different types of EVs by their priorities. However, the issue of maximum and minimum green period in traffic engineering is not discussed in this article. Qin et al. proposed an improved algorithm that reduces response time and minimizes the effects of general traffic by using two control strategies [8]. The system changes the traffic state to EVSP transition 1 to reduce the traffic impact time, after transition 1, the system switches to EVSP transition 2 for traffic flow compensation.

### 2.2. Road Side Unit (RSU)

Kuo et al. proposed a new traffic control system for communication policy management, communication management, remote management, and developed communication protocols among RSU, OBU, and the cloud [9]. Al-Dweik et al. [10] proposed a scalable enhanced roadside unit, the main components of which are the speed adaptive traffic control system, the pollution adaptive traffic control system (PATC), weather information system, and master control center (MCC). It aims to improve traffic flow by opening or closing specific routes through PATC and use MCC to monitor weather, coordinate road maintenance services, and drive and maintain in bad weather.

Kantawong et al. designed smart traffic cones to communicate with vehicles by using RFID, they combined with image compression analysis algorithms to develop a new traffic cone vehicle accident detection and recognition system [11]. This paper uses wavelet transform image compression with vector quantization. Sanghyun Ahn et al. proposed a new mechanism by selecting the vehicles performing V2I communications based on the concept of road sectorization [12]. This mechanism can effectively reduce transmission overhead. In practice, the vehicle sends a message packet in a specific area. The message packet may be lost due to environmental factors, and the traffic signal controller does not receive the information of the vehicle. Fogue et al. [13] proposed an e-Notify system architecture system that uses sensors (such as airbag status, throttle status, brake status) and algorithms to determine if an accident has occurred. When the system detects a vehicle has a traffic accident, it broadcasts the message to the RSU. RSU receives traffic accident information and uploads information to the cloud platform via mobile communication.

### 2.3. Adaptive Traffic Signal Control (ATSC)

Pandit et al. [14] proposed an adaptive traffic signal control with the Oldest Arrives First (OAF) strategy by using a vehicular network (802.11p). The OAF algorithm controls the traffic with two phases where Phase I platoons the vehicles using the real-time vehicle positions, it first maximizes the size of the platoon, and then minimizes the size difference of platoons. In Phase II, OAF schedules each platoon to pass the intersection by the oldest arrives first principle. The simulation results show that the OAF algorithm has the best performance compared with traditional traffic signal scheduling algorithms, including the pre-timed, vehicle actuated logic, and Webster methods [14]. Hossam et al. presents a novel de−centralized flexible phasing scheme, where the traffic signal controller uses a Nash bargaining game−theoretic framework [15]. Although the proposed algorithm is innovative, the lack of some traffic protection measures, such as maximum green time and minimum green time, may lead to unsafe cases for pedestrians.

Some well-known ATSC models are commonly adopted in urban intersections, as illustrated in Table 1, including: SCOOT [16], SCAT [17], RHODES [18], COMDYCS III [19], LHOVRA [20], and OPAC [21]. The common issue is that the spatiotemporal coverage of traffic information is not enough for optimal control, and the costs for construction and maintenance of the loop detector are high.

### 2.4. V2X-Based Eco-Driving

Lee et al. proposed two eco-driving models, the maximum throughput model (MaxTM) and the minimum acceleration and deceleration model (MinADM) [22]. This work shows that the performance of carbon emissions is 5–102% better than MinADM and 13–209% better than the compared baseline (OTLCM [23]). In real traffic cases, MaxTM is 8–14% better than MinADM and 15–231% better than OTLCM. Lee and Li extended eco-driving models in [22] from standalone intersection to multiple intersections, an energy-saving driving advisory system (EDAS) was introduced [24]. OBU calculates the speed range for passing through at most four intersections based on signal plans broadcasted by RSU. Two strategies including the maximum throughput model (MTM) and smooth speed model (SSM) are designed and being adopted according to the degree of traffic congestion. Experimental results show that the MTM of the single intersection travel time is 20.5–84.3% less than the OTLCM. The carbon emissions of continuous intersections MTM+SSM are 19.9–31.2% less than predictive cruise control (PCC) model [25], and MTM+SSM is 24.5–35.9% less than PCC in travel time.

The discussions of literature related to eco-driving models including concepts, relationship between RSU and OBU, benefits, and applications are summarized as in Table 2.

## 3. Smart Traffic Control System

### 3.1. STSC System Architecture

**Figure 1:**
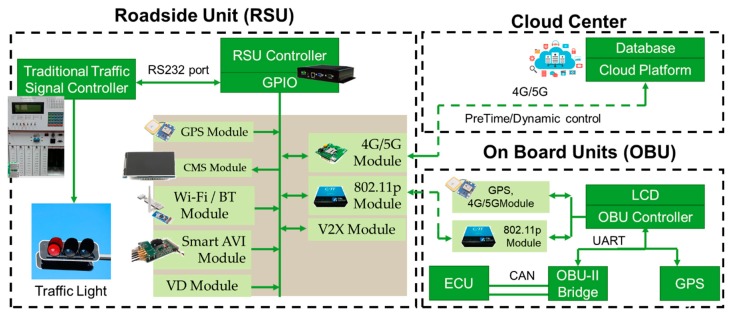
System architecture of the STSC system.

The architecture of the proposed STSC system is shown in Figure 1, and it is composed by three subsystems including an RSU, an OBU, and a cloud center. The RSU controller, connected to traffic signal control system, is the key component in STSC. It is implemented by an industrial computer designed by modularization concept where multiple external modules can be flexibly added onto the RSU by the general−purpose input/output (GPIO) interface. The supporting peripheral modules includes 802.11p V2X interface, GPS, changeable message sign (CMS), mobile communication (4G), and Wi-Fi/BT modules. In addition, the RSU has more GPIO for real-time traffic information extension, such as loop detector or video traffic analyzer (smart AVI).

On the OBU side, it is implemented by an on-board computer which connects to several modules including 802.11p V2X interface, GPS, mobile communication (4G), and OBD-II interface. The OBD-II interface can read the vehicle′s information such as oil temperature, engine speed, speed, turn signal, etc. The cloud center is designed by a cloud platform and a central database which provides management functions to all the RSUs and OBUs.

RSU periodically broadcasts the signal plan (cycle time, green split, current phase, current countdown) and events (incident, road work warning) using an 802.11p interface, vehicles equipped with OBU can receive and react to the real-time information, such as eco-driving suggestions, path re-routing. On the other hand, emergency vehicles (ambulance, police vehicle) equipped with OBU will periodically broadcast the current status using a V2X interface, and an RSU within the signal coverage range can collect the EV messages and do instant responses.

**Figure 2:**
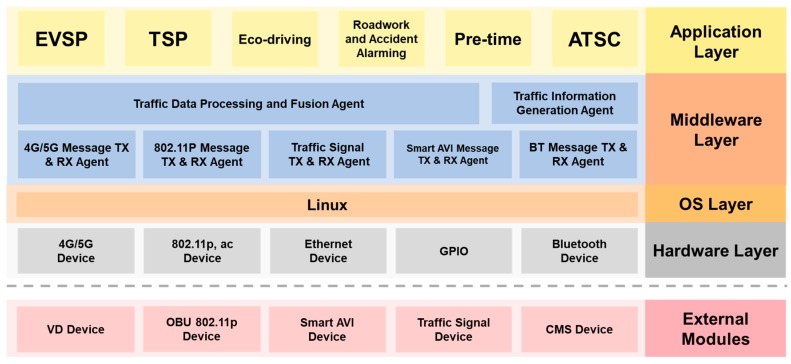
Software architecture of the RSU controller.

In order to fast and cost−effectively deploy the STSC system, our idea is that the RSU controller has to be compatible with the existed infrastructure so that there is no need to replace the traditional traffic controllers. The proposed RSU controller should easily connect to and control the legacy signal controller, regarding it as one of the peripheral devices. To achieve this goal, a modularized system software architecture is designed as shown in Figure 2, which is a five-layered hierarchical structure of the RSU controller.

An application layer, which is the pool of the application programs, supports six smart city applications including emergency vehicle signal preemption (EVSP), transit signal priority (TSP), eco-driving by signal plan broadcasting, roadwork and accident warning, pre-time signal control, and adaptive traffic signal control (ATSC). Middle layer is the kernel of the RSU, where seven important agents are designed. Traffic data agent and traffic information generation agent are responsible for the data processing and data fusion collected from external data sources (loop detector and smart AVI) to generate real-time traffic information for ATSC. Five message TX/RX agents are designed for dealing with message transmitting and receiving on five device channels, where buffers, queues, and priority mechanisms are designed to handle the different kind of events and messages from other agents. The operating system layer is based on Linux OS and the hardware layer is the driver layer for multiple hardware access interfaces. The external modules handle the input/output interfaces for accessing different external modules, such as CMS module, Smart AVI module, 802.11p modules.

### 3.2. STSC State Transition

**Figure 3:**
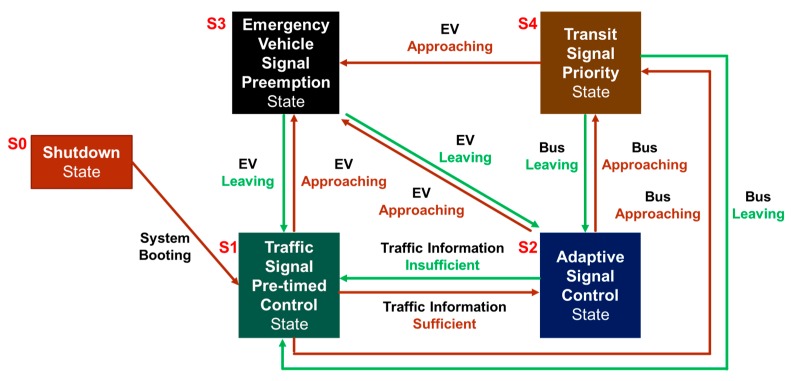
Smart traffic signal controller’s state transition diagram.

In order to integrate multi-modal transportation applications in an RSU controller, a five-status state transition diagram for the RSU controller is designed as illustrated in Figure 3, where the state transition condition and control flow of the five states (S0, S1, S2, S3, S4) are described.

The RSU controller switches from shutdown state (S0) to traffic signal pre-timed control state (S1) when the system is started up, the traffic signal control follows the predefined time−based signal plan optimized by historical data which is assigned from the cloud center. It switches to ATSC mode (S2) when the RSU has enough real-time traffic information collected from external data sources (such as loop detector, smart AVI), a special designed adaptive traffic signal control algorithm will be responsible for the traffic flow control optimization in this mode.

EVSP (S3) state has the absolute priority since emergency vehicles has the non-stop authority over the traffic signal control when the EVs are on duty. When RSU detects the OBU packets from EV, it switches to EVSP state (S3) as soon as possible, despite which mode it is currently on. Comparing to absolute priority strategy applied in EV, the public transportation has the relative priority, so that an independent TSP mode (S4) is designed for public transit signal priority because the signal control algorithm of TSP is very different from the one in EVSP mode.

Usually the RSU operation is mainly on S1 or S2 mode depending on whether the real-time traffic information is enough or not. When the RSU controller receives the messages from an emergency vehicle or a transit signal priority vehicle (such as BRT, bus), the state transition control algorithm will first determine whether it is the host RSU (i.e., the responsible intersection) by comparing the location, direction, speed message collected from the vehicle and the location of itself. It then switches to S3 and begins the EVSP process or S4 for the TSP process if it is the host RSU. In these two modes (S3 or S4), the RSU controller will monitor the status of the vehicle from the vehicle-to-RSU (V2R) packets until it already passes through the intersection; it then begins the signal compensation process and switches to the original operation mode (S1 or S2).

### 3.3. Emergency Vehicle Signal Preemption Scenario

**Figure 4:**
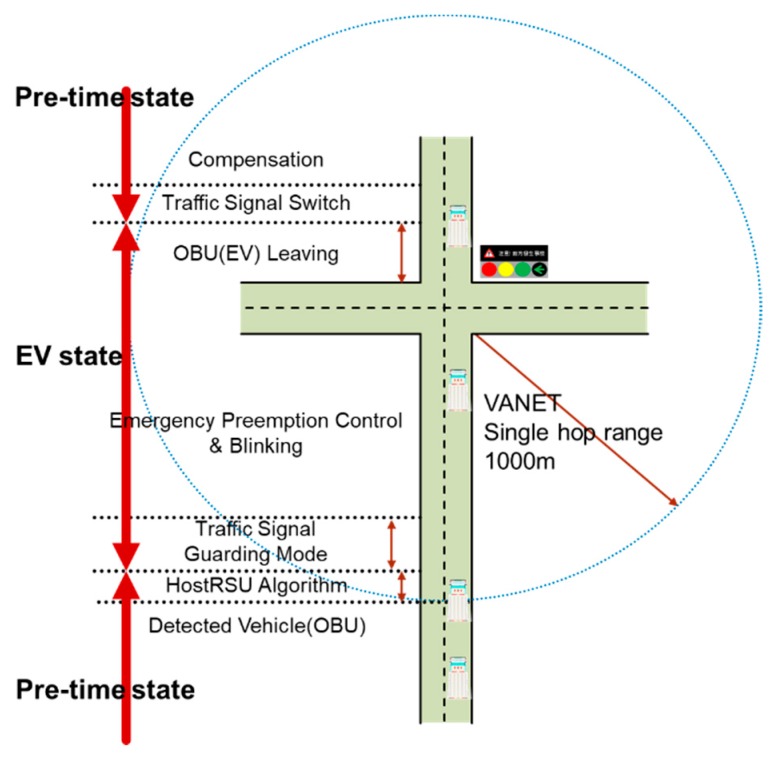
Emergency vehicle signal preemption (EVSP) scenario.

To illustrate the STSC system process, we take the EVSP scenario as an example to discuss in detail the state transition, control algorithms, and information flow among STSC system components. As shown in Figure 4, the EVSP scenarios have seven stages and two state transitions from RSU detecting approaching EV to EV leaving. RSU switches from pre-time mode to EVSP mode if it is the host RSU. The RSU then begins the whole EVSP processes and switches back to pre-time mode after traffic signal compensation, which is designed for compensating the crossing traffic flows stopped by signal preemption.

In Figure 4, assuming an EV is on duty and approaches to the intersection from the south, the RSU receives the EV message broadcasted from the OBU. Once the EV entering the 802.11p signal coverage range, seven stages will be done to complete the emergency vehicle signal preemption process including detected EV, determined host RSU, traffic signal guarding mode, EV preemption control, EV leaving, signal switch, and traffic flow compensation, then the system state switches back to the original state. Each phase has a specific control algorithm to deal with, which are discussed in the following sections.

## 4. EVSP Process

**Figure 5:**
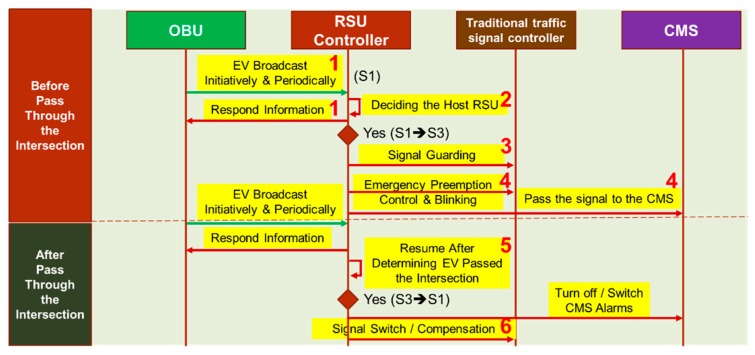
Sequence diagram of the EVSP mode.

As shown in Figure 5, four components are interacted in the EVSP sequence diagram, which are OBU, RSU controller, traffic signal controller, and CMS. The interaction messages sequence can be divided into two phases, before and after the EV passed through the intersection, which are mapping to the seven stages illustrated in Figure 4. The RSU controller collects the EV message, decides if it should be responsible for the EV (host RSU), controls the signal control, and displays messages on the CMS. After EV passes through the intersection, the RSU controller switches back the signal, CMS, and starts traffic compensation as discussed later in Section 4.5.

**Figure 6:**
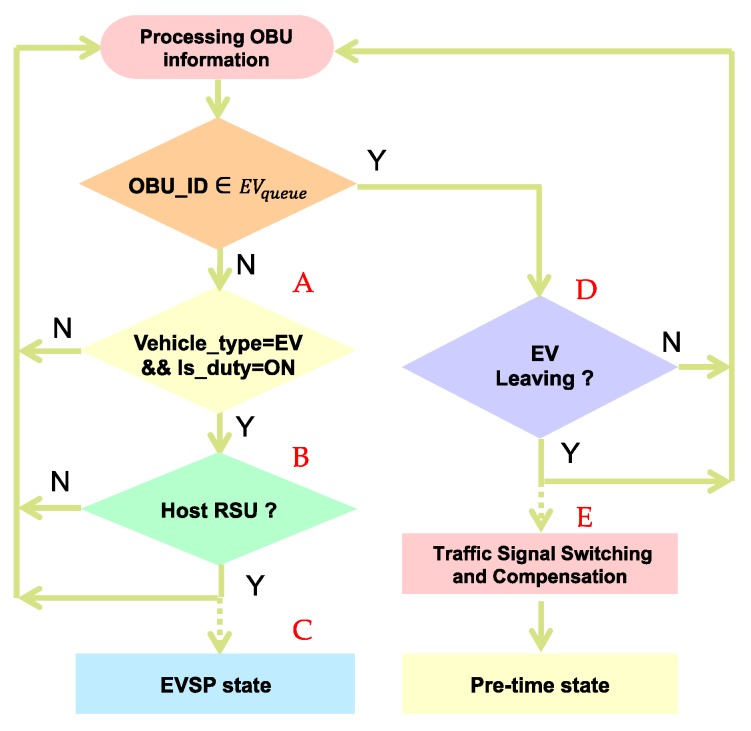
EVSP algorithm.

The major component where the EVSP control algorithm executed is the RSU controller, where the system flowchart is shown in Figure 6. The EVSP algorithm processes the OBU message, identifies the vehicle type and status (A), determines if it is the host RSU by the HostRSU algorithm (B), entering the EVSP state, and running the traffic signal control algorithm (C), determining if OBU leaves the intersection (D), and switches to the compensation mode (E).

EV broadcasts an on-duty preemption message, RSUs which received the message will determine if it should be responsible for this EVSP message by the host RSU algorithm (discussed in Section 4.2). RSU switches to S3 (EVSP mode) if it is the host RSU, and activates the preemption process and signal guarding mode (yellow + all red). It displays EV information on the CMS to inform all the drivers and passengers. The RSU controller continuously monitors the location and status of the EV, and determines if it has passed through the intersection. When the EV has left the intersection, it switches the state from S3 to the original state, turns off the CMS message, and begins the traffic compensation process.

### 4.1. Vehicular Network Communication Protocol

**Figure 7:**
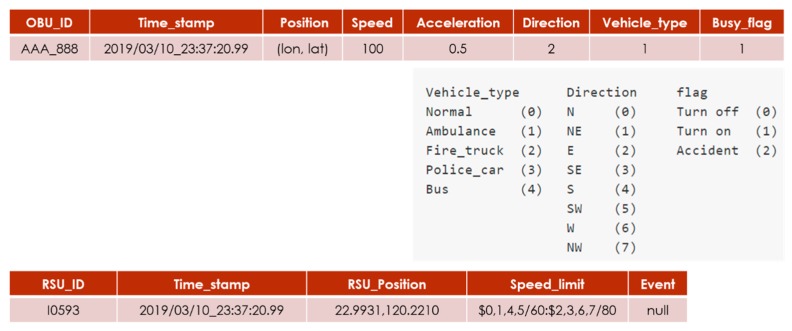
Vehicular network communication protocol.

The STSC system communication protocol defines the message, format, frequency between the OBU, and the RSU, as shown in Figure 7. OBU periodically broadcasts the vehicle-to-RSU (V2R) message every second, the message fields are OBU_ID, TIME_STAMP, POSITION, SPEED, DIR, ACC, VEHICLE_TYPE, DUTY_FLAG, where time, position, and speed information collects from GPS module, id, type, and duty are the vehicle internal status. The DIR field determines the direction range (from 0–7), converted from the GPS data, is applied for matching the vehicle direction and RSU location, which is applied for the host RSU determination algorithm. DUTY_FLAG indicates whether the emergency vehicles is on duty or not.

RSU periodically broadcasts an RSU-to-vehicle (R2V) message every second, where message fields including RSU_ID, TIME_STAMP, RSU_POSITION, SPEED_LIMIT, and EVENT, represents unique RSU ID, timestamp, the location of the RSU, speed limit, and warning events (ex. accident, EV, road work), respectively.

### 4.2. HostRSU Algorithm

**Figure 8:**
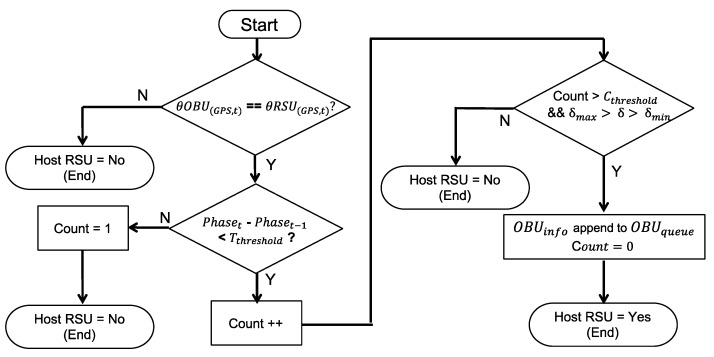
HostRSU algorithm.

The HostRSU algorithm determines whether the RSU itself should be responsible for the approaching emergency vehicle. As shown in Figure 8, when the RSU receives a message, it first checks the EV approaching angle ($\theta OBU_{\left(GPS,\;t\right)}$) and determines if it falls within the RSU service angle. The message will be ignored if the moving angle of the approaching EV does not fit into the RSU service range, which means that this EV may not move on sections of this intersection. The algorithm then double confirms the EVSP request by checking if the received OBU packets is more than a threshold ($C_{threshold}$). The third check is the service range: the distance from the vehicle to the signal controller (δ) should fall into the service range predefined in the RSU; otherwise, it ignores the message. The RSU confirms it is the host RSU only if all these checks are passed, it then returns true and starts the EVSP process.

### 4.3. Traffic Signal Control Algorithm

**Figure 9:**
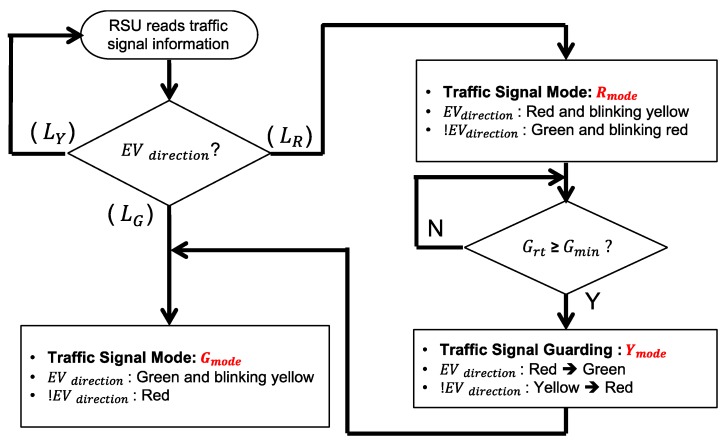
Traffic signal control algorithm.

After the host RSU check, the RSU starts the EV signal preemption control process, where the control algorithm of this process is presented in Figure 9. RSU reads the current signal plan from the traffic signal controller, determining whether to extend the green period or cut off the red period to facilitate the EV passing through the intersection. The green mode (*G_mode_*) indicates that the EV direction is currently in the green period and that it should remain green until the EV passes the intersection. If the EV direction is currently in red mode (*R_mode_*), the signal controller should turn to green as soon as possible but still has to follow the minimum green constraints to ensure the safety of pedestrians.

A traffic signal guarding mode (*Y_mode_*) is designed after the *R_mode_* control is completed. The RSU judges the remaining green time $(G_{rt}$) of the signal in the non-EV direction. If $G_{rt}$ is smaller than minimum green protection time $\left(G_{min}\right)$, $R_{mode}$ will continue until $G_{rt}$ is greater than $G_{min}$. It then enters the traffic signal guarding mode (*Y_mode_*), where the RSU will convert the non-EV traffic light from yellow to red. The RSU controls the signal into green mode (*G_mode_*) after *Y_mode_*, and stays in *G_mode_* until the EV leaves.

**Figure 10:**
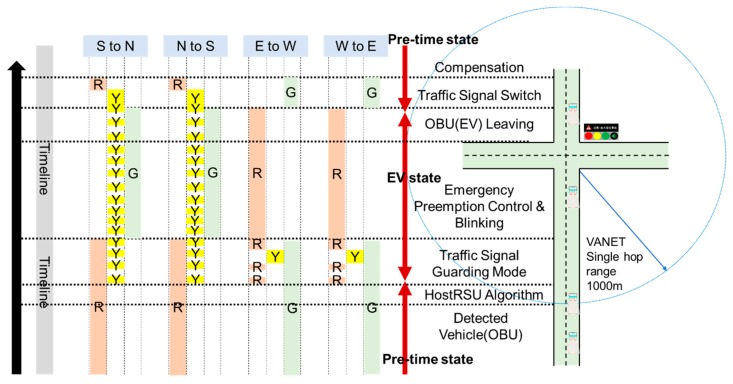
Traffic signal blinking mode $\left(R_{mode}\right)$.

**Figure 11:**
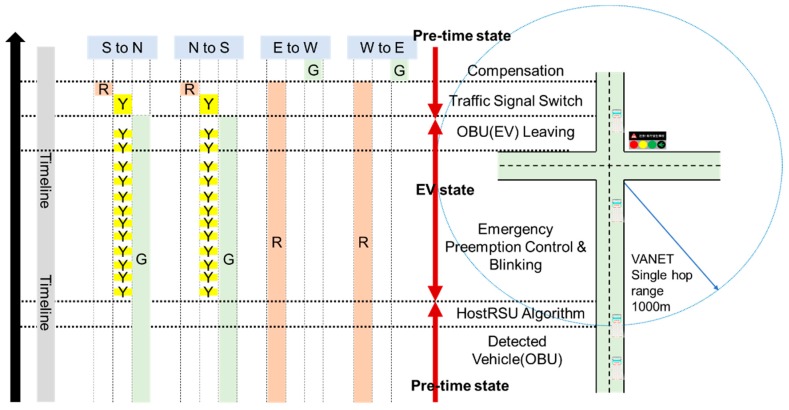
Traffic signal blinking mode $\left(G_{mode}\right)$.

A new traffic signal mechanism is specially designed for smoothing the EVSP system operation. It is constituted by two traffic signal blinking modes: *R_mode_* and *G_mode_*, as shown in Figure 10 and Figure 11, where *R_mode_* is responsible for the handling the process of signal control when the signal of EV direction is red, and *G_mode_* is responsible for the condition that signal is green. Different from the traditional single green/yellow/red light, a hybrid signal scheme is designed to notify all the nearby vehicles and pedestrians that an EV event is now happening and provides information on which direction the EV is coming from. For example, the signal will display red with blinking yellow at the same time in the EV direction to inform all vehicles where the EV is coming, and the signal will change to green as soon as possible, and switches to green with blinking yellow to facilitate EV going through the intersection. A changeable message sign (CMS) attached on the signal displays a message to inform from which way the EV is coming, and which way the vehicles should move to facilitate EV through and ensure safety.

• Traffic Signal Mode: *R_mode_*

In *R_mode_*, where the current signal in the EV direction is red, RSU will begin the process of switching the signal to green as soon as possible to facilitate EV moving through the intersection, while the process still has to follow a minimum green constraint to ensure safety.

The process of the *R_mode_* scenario is illustrated in Figure 10. Assume that the EV is on emergency duty and approach the intersection from the south direction. If the signal is in a red period, the RSU will activate the *R_mode_* procedure after it determines it is the host RSU. The red period will be cut off and smoothly switched to green due to the EV owning the priority. The traffic signal control algorithm controls the signal and displays red and blinking yellow (instead of the red) in EV moving directions, and green and blinking yellow (instead of green) in cross directions. This signal scheme as well as the message shown in CMS are specially designed to inform all the vehicles and passengers from which direction the EV is coming and how to move for giving way.

Another important issue is that the minimum green constraint (*G_min_*) should be followed for the pedestrian protection; this period is the traffic signal guarding mode shown in Figure 10. After traffic signal guarding, the control algorithm switches to the *Y_mode_*, which includes a short blinking yellow followed by an all red interval, which is the interaction clearance interval for clearing the intersection to ensure safety. In EV preemption control and blinking stage, the algorithm controls the signal in EV directions with green and blinking yellow to signal to other vehicles, and red for crossing directions to stop vehicular conflict. Once the RSU detects that the EV is leaving the intersection (discussed later in Section 4), it switches back the signal and conducts the compensation process (discussed in Section 5). 

• Traffic Signal Mode: *G_mode_*

In *G_mode_*, the traffic signal in EV directions is originally green, the task of the control algorithm is to maintain or extend the green period, clearing the vehicles in front of the EV to facilitate it moving through the intersection. The signal control process of *G_mode_* is illustrated in Figure 11, it extends the current green phase and controls the signal in green and blinking yellow in EV directions (S to N, N to S), and stays red in crossing directions (E to W, W to E). The extending green period that should be compensated after EV leaves the intersection is discussed in Section 4.5.

### 4.4. OBU Leaves the Intersection Process

**Figure 12:**
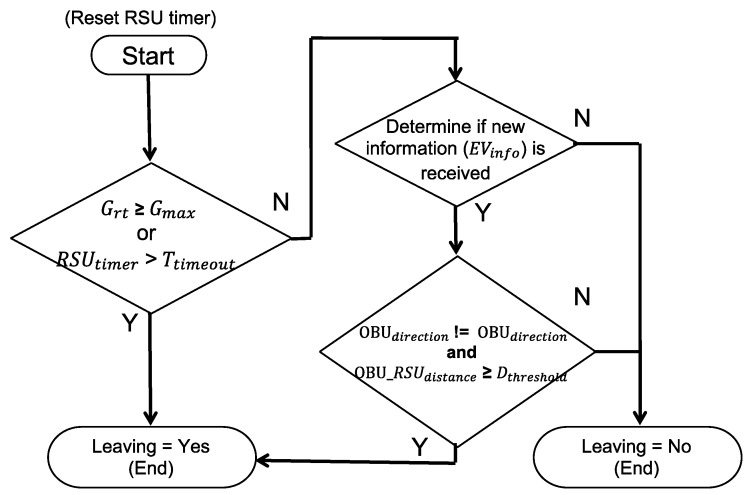
The algorithm of determining if OBU leaves the intersection.

When the RSU is in EVSP state, it continuously monitors the state and position of the EV and determines if the EV leaves the intersection. As shown in Figure 12, the RSU starts a timer (*RSU_timer_*), and continuously monitors if it is timeout or violates the maximum green constraint. The purpose of the timer is to prevent the RSU from staying in an EVSP state if information broadcasted by OBU without being received after EV leaves the intersection.

### 4.5. Traffic Signal Switching and Compensation Mode

**Figure 13:**
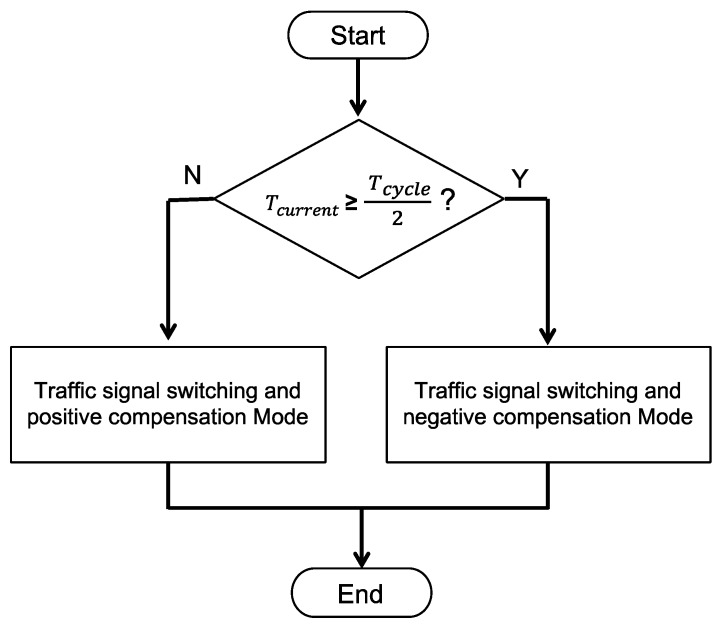
Traffic signal switching and compensation mode.

After the EV has passed through the intersection, the RSU switches back to original signal phase and starts the traffic flow compensation. The purpose of the compensation mechanism is to improve the vehicle’s continuation rate and reduce the unnecessary stagnation. Two compensation modes including positive compensation and negative compensation are designed, as shown in Figure 13, which enables the proposed STSC system to reduce the time of the signal coordination and is backward compatible with legacy traffic signal controllers. The RSU will switch to negative compensation mode if the current signal phase is more than half; otherwise, it switches to the positive compensation mode.

### 4.6. Multiple Emergency Requests

A conflict requests handling mechanism is designed to deal with the case when multiple prioritized vehicle requests are received at the same time. If EV and TSP requests are received in RSU at the same time, EV requests should be handled as prioritized rather than TSP requests because EV has the authority of signal preemption. In the case of multiple EV requests from different approaches, we give emergency vehicles (ambulances, fire engines, police cars) different weights. For example, if the weight of ambulance is higher than the police vehicle, RSU will give priority to the ambulance service when both requests are received at the same time. The first-come first-served (FIFO) operation strategy is adopted if two EV requests from different approaches received the same weight.

## 5. System Implementation and Experiment Results

In this section, the design and implementation details of the STSC system are discussed. The integration of the RSU controller and traffic signal controller is introduced, and the integration test is presented. Unit and functional test of OBU and RSU communication, and demonstration of the field experiment in EVSP are presented in this section.

### 5.1. STSC System Implementation

**Figure 14:**
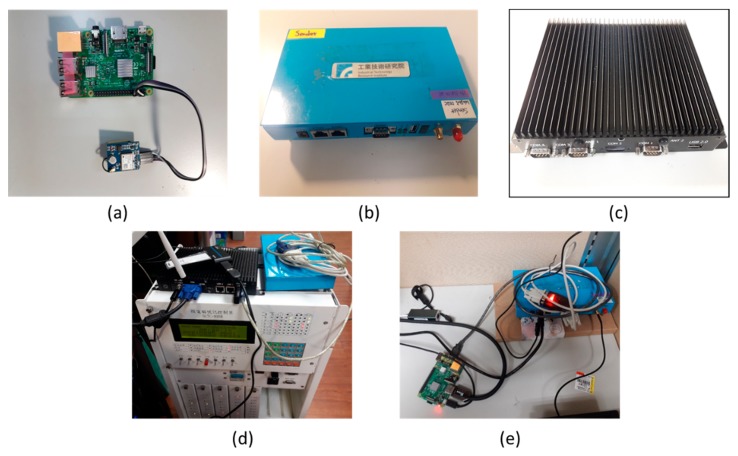
STSC system hardware implementation: (**a**) OBU embedded computer, (**b**) 802.11p module, (**c**) RSU controller, (**d**) RSU prototype system, (**e**) OBU prototype.

OBU is implemented by an embedded computer (Raspberry Pi 3), which connects 802.11p, GPS modules, and a small LCD screen for display messages, as shown in Figure 14a,e. The 802.11p communication module (IWCU V4.2) developed by the Industrial Technology Research Institute (ITRI), as shown in Figure 14b, is used for V2X communication. The hardware interface of the module includes two PCIe gigabit ethernet interfaces, one RS232 port, and two USB2.0 ports. It is a Linux−based operating system shipped with a software development toolkit and a cross compiler. In the STSC system, we adopt the 802.11p module in both the RSU controller and the OBU equipped in the EV.

A small industrial computer is adopted as the RSU controller (SEM−6338), as shown in Figure 14c, where the main components in it includes embedded SOC (AMD^®^ G-Series), 8G DDR3 ram, 2 RS232 ports and 1 RS232/422/485 Port, an ethernet interface and four USB ports. Through a variety of hardware interfaces, the RSU controller can effectively access the data of external peripheral modules. The operation system of the RSU controller is Linux Ubuntu version 18.04. Several peripheral devices are connected to the RSU controller including 802.11p module, traffic signal controller (as shown in Figure 14d), CMS module, 4G module, and Bluetooth/Wi-Fi module.

In order to quickly and cost-efficiently deploy the proposed STSC system, one key successful factor is that it should be compatible with legacy traffic signal controllers. The RSU controller can connect to a legacy traffic signal controller as shown in Figure 14d and is designed following the Metropolitan Traffic Control Protocol V3.0 national standard defined by the Ministry of Transportation and Communication (MOTC). In this work, the signal controller manufacturer modified the firmware to support the special designed signal scheme so that the RSU controller can issue more control commands to the signal controller, such as green and blinking yellow. By this approach, the proposed STSC can be compatible with the legacy traffic signal controller and it can be quickly deployed for multi-modal smart city ITS applications.

### 5.2. V2R Module Test

To test the communication range of the vehicular communication between OBU and RSU, two 802.11p modules are used for the receiving and the transmitting test. The receiving end is fixed at a point which acts as the role of RSU controller, and the transmitting end placed at a distance from the RSU is set up for evaluating the V2R/R2V communication range. The test communication distance ranges from 25 m to 400 m, and the transmission signal strength (in dB) and received signal strength (in RSSI) are listed in Table 3. The evaluation result shows that the distance of OBU and RSU that falls within 400 m can exchange data correctly.

### 5.3. RSU Controller and Signal Controller Integration

The RSU controller connects to a signal controller and periodically reads the traffic signal plan which contains the information of signal state, phase separation, steps, seconds, and control strategy. The RSU controller can send commands to the signal controller such as extending the green period or cutting off the red period. The RSU controller periodically broadcasts the real-time signal plan via 802.11p or Bluetooth/Wi-Fi interface, OBU or smart phone App in the radio signal coverage range can receive the signal plan data and reacts to the signal plan. For example, vehicles can take action before the signal change and performs eco-driving or re-routing.

**Figure 15:**
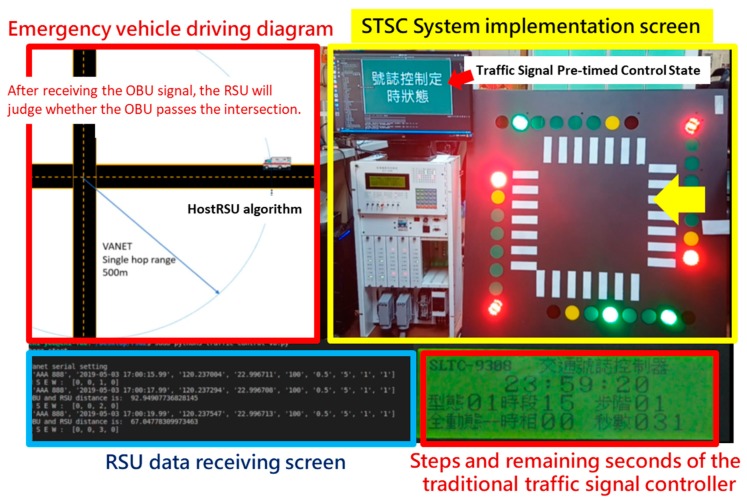
Testing EVSP functions in the lab.

An integration validation experiment is performed using an EVSP scenario, as shown in Figure 15, where a LED signal simulation board is attached to the signal controller as simulating the control output of the designed control algorithm. We simulate an EV approaching the intersection, and the message broadcast by OBU is collected by RSU and displayed on the screen (lower left part), and the EVSP control algorithm sends the command to a signal controller to execute the whole EVSP processes (lower right part).

Some module verifications in the RSU are performed including the state transition test, the host RSU determination algorithm, the control verification of the signal in the EVSP state, and the function of judging the departure of the emergency vehicle. The function verification in the OBU includes the control of GPS and 802.11p modules, and continuously broadcasts information at a fixed frequency (1 Hz). We also did a detailed test on the function of HostRSU determination test, including the long-time processing mechanism, the distance threshold judgment mechanism, the phase angle matching mechanism, and the multiple emergency vehicle request determination tests.

### 5.4. EVSP Scenario Demostration in Field

**Figure 16:**
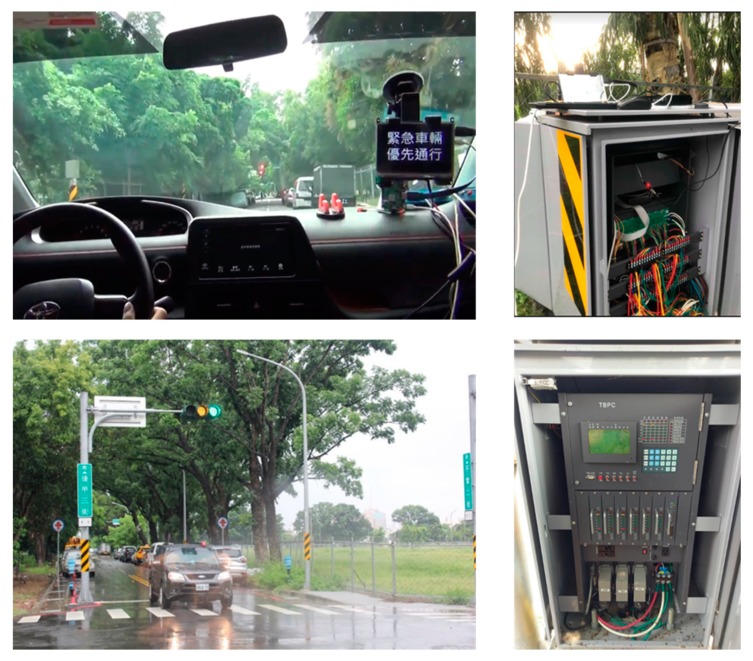
Pictures of Field test for the EVSP application (in Pingshi Park).

After the module test and integration test, an EVSP field test is done to verify the proposed STSC system. One signalized intersection in Pingshi Park in Tainan city is chosen for the EVSP field test. We cooperate with the domain experts from the Transportation bureau of Tainan city government to evaluate the EVSP scenarios. The test results are successful, both in the green mode and red mode cases when EV approaches the intersection. Some pictures of this EVSP field test are shown in Figure 16, and the field test video hyperlink is attached on the Supplemental Materials.

## 6. Conclusions and Future Works

In this paper, a multi-modal smart traffic control system (STSC) for the infrastructure in the smart city is proposed, which can be widely applied for the intelligent transportation system in smart city applications. The major components in the proposed STSC include RSU controller, OBU, signal controller, and cloud center. It supports various smart city ITS applications including EVSP, TSP, eco-driving, ATSC, pre-time signal control, and R2V message broadcasting.

The RSU controller is the core of this work, where we discuss in detail the system architecture, middleware, peripheral hardware modules, and control algorithms. The STSC system designed follows the urban traffic control protocol V3.0 so that it is compatible with a traditional traffic signal controller and can be fast and cost-effectively deployed. A new traffic signal scheme is specially designed for the EVSP scenario; it can inform all the drivers near the intersection when EV is approaching, smoothing the traffic flow and enhancing the safety without extra hardware costs.

In the future, real-time traffic information fusion for ATSC by integrating multiple data sources such as loop detector and smart AVI is to be designed. Design details for TSP and ATSC applications should be further implemented. Kernel optimization in middleware modules such as message handling, state transition, and parallel processing are planned to be implemented. Cooperated RSU controllers for optimizing EVSP, TSP, or ATSC can be further studied to enhance the efficiency of public transportation.

## Figures and Tables

**Table 1:** Adaptive traffic signal control models.

ATSC Model	Sensor	Sensor Location	Control Strategy
**SCOOT [16]**	Loop	Upstream 10~20 m	Minimize the sum of the averaged queue
**SCAT [17]**	Loop	Stop line	Minimize stops and travel time
**RHODES [18]**	Loop	Upstream of the stop line	OD matrix, flow control, and signal control
**COMDYCS III [19]**	Loop	Two detectors: 0 m and 150~300 m from stop line	Multiple optimization objectives
**LHOVRA [20]**	Loop	10 m, 85 m, 140 m, 200 m, 300 m from stop line	Multiple optimization objectives
**OPAC [21]**	Loop	upstream 400~600 feet	Maximize the sum of performance indices on all approaches

**Table 2:** Eco-driving models.

Eco-Driving Model	Concept	Actions	Benefit	Application
**MaxTM, MinADM [22]**	Max. throughput and Min. acceleration and deceleration	OBU suggests eco-driving speed	Reduce carbon emissions, fuel consumption, travel time	Standalone intersection
**VANET−based coordinated signal control model [26]**	Forecasting and decision making	RSU determines traffic signal plan	Reduce carbon emissions and fuel consumption	Multiple intersections
**EDAS System [24]**	Calculate the number of intersections that can be passed and take different modes	OBU suggests eco-driving speed	Reduce carbon emissions, fuel consumption, travel time	Multiple intersections
**TTE&RS [27]**	Travel time prediction and path recommendations	OBU forecasts travel time and suggests path	Reduce computational complexity and reduce travel time	Multiple intersections

**Table 3:** Communication distance test of 802.11p module.

Distance	Signal Transmission Strength (dB)	Signal Receive Strength (RSSI)
25 m	12	−79
50 m	15	−77
80 m	15	−86
100 m	15	−89
150 m	15	−88
200 m	18	−92
265 m	22	−93
300 m	22	−92
400 m	25	−87

## Supplementary Materials

EVSP filed test video, which can be browsed through online video platform at the URL: https://www.youtube.com/watch?v=CvT5rPOm4I0.
